# The goals of care in acute setting for geriatric patients in case of a hip fracture

**DOI:** 10.1007/s00068-023-02258-0

**Published:** 2023-03-18

**Authors:** Thomas Marcus Paulus Nijdam, Duco Willem Pieter Marie Laane, Tim Ellen Eloeska Schiepers, Diederik Pieter Johan Smeeing, Diederik Hendrik Ruth Kempen, Hanna Cunera Willems, Detlef van der Velde

**Affiliations:** 1 Department of Trauma Surgery, St. Antonius Hospital, Utrecht, The Netherlands; 2 Department of Trauma Surgery, University Medical Center Utrecht, Heidelberglaan 100, 3584 CX Utrecht, The Netherlands; 3 Department of Orthopedic Surgery, OLVG, Amsterdam, The Netherlands; 4 Department of Internal Medicine and Geriatrics, Amsterdam University Medical Centers, Amsterdam, The Netherlands

**Keywords:** Trauma surgery, Hip fracture, Geriatric, Shared decision making, Goals of care

## Abstract

**Purpose:**

For geriatric hip fracture patients, the decision between surgery and palliative, non-operative management is made through shared decision making (SDM). For this conversation, a physician must be familiar with the patient’s goals of care (GOC). These are predominantly unknown for hip fracture patients and challenging to assess in acute setting. The objective was to explore these GOC of geriatric patients in case of a hip fracture.

**Methods:**

An expert panel gathered possible outcomes after a hip fracture, which were transformed into statements where participants indicated their relative importance on a 100-point scoring scale during interviews. These GOC were ranked using medians and deemed important if the median score was 90 or above. Patients were aged 70 years or older with a hip contusion due to similarities with the hip fracture population. Three cohorts based on frailty criteria and the diagnosis of dementia were made.

**Results:**

Preserving cognitive function, being with family and being with partner scored in all groups among the most important GOC. Both non-frail and frail geriatric patients scored return to pre-fracture mobility and maintaining independence among the most important GOC, where proxies of patients with a diagnosis of dementia scored not experiencing pain as the most important GOC.

**Conclusion:**

All groups scored preserving cognitive function, being with family and being with partner among the most important GOC. The most important GOC should be discussed when a patient is presented with a hip fracture. Since patients preferences vary, a patient-centered assessment of the GOC remains essential.

## Introduction

The prevalence of hip fracture is increasing due to ageing of the Western population [1, 2]. Hip fracture patients are most commonly treated with operative management (OM) for early mobilisation and rehabilitation [3–5]. For geriatric hip fracture patients with limited life expectancy, palliative, non-operative management is an alternative option, which aims not to restore mobility or independence, but to focus on palliative care and pain management [5–8]. Recently, it has been shown that this non-operative option is non-inferior to OM in terms of quality of life for geriatric patients with limited life expectancy [7]. Since palliative, non-operative management is a viable treatment option, physicians have to discuss it in the acute setting through shared decision making (SDM) [5]. This decision-making process starts with the identification of the patient’s goals of care (GOC) which enables a tailor-made SDM discussion considering the patient clinical and personal needs [5, 9–14].] These GOC can vary significantly between patients. For example, the urge to rehabilitate or undergo life-prolonging interventions may not be the same for every geriatric patient sustaining a hip fracture. For oncological and chronic diseases, patients’ future palliative needs can be discussed at earlier timepoints before the disease reaches an end state [15]. On the contrary, geriatric trauma patients and family members have not thought about them before being confronted with palliative treatment options at the emergency department. Furthermore, the hip fracture patient usually are unknown at presentation to trauma physicians without previous treatment relationships, which makes identification of the GOCs challenging in the relative short time window to surgery [16–19]. Since advance care planning (ACP) is often still lacking, patients have not explicitly considered their GOCs in case of a hip fracture [5, 20]. Exploring general GOCs of hip fracture patients could help physicians to discuss and assess the individual preferences for future patients at the Emergency Department (ED). Therefore, this study aims to investigate the most relevant GOCs of geriatric patients in case of a hip fracture.

## Materials and methods

### Study design

A cross-sectional survey study was conducted to obtain important GOC for geriatric patients in hip fracture setting. Patients with a hip contusion were deemed as most appropriate study population, because this population is similar in characteristics to hip fracture population but cannot have been influenced by experiences with OM [21]. Patients were eligible for inclusion when diagnosed with a hip contusion at the ED of a large regional teaching hospital between January 1, 2021 and September 1, 2022. The study design was approved by the Medical Ethics Committee, Utrecht (MEC-U), The Netherlands (W22.149). The Checklist for Reporting of Survey Studies was used to guide this article [22].

### Survey design

The survey was constructed using Passmore’s guidelines [23]. To complement the survey, an expert panel (consisting of a trauma surgeon, orthopedic surgeon, clinical geriatrician and a nursing home physician) was consulted to identify additional desirable and undesirable outcomes associated with a hip fracture. After the experts had provided possible outcomes, the results were combined into a list. All suggested outcomes were reviewed and consensus was reached between the experts. These outcomes were transformed into statements, which were organized into three themes: treatment, rehabilitation and quality of life (QoL). To ensure validity, the statement “Length of life is more important than quality of life” was added which should be scored oppositely to the statement “Maintain quality of life (is more important than prolonged life)”. As a final question, participants were asked if important outcomes not mentioned during the interview were missing. All participants received similar information before conducting the interview. The structured interview is attached as Appendix 1. Pretesting on completeness and understanding of the interview was performed by conducting the interview in three patients aged 70 years or above who were admitted to the trauma geriatric ward with a hip fracture. The pre-test patients were deemed similar to the sample population. All pre-test patients indicated the statements were comprehensible and complete.

### Participants

Patients were eligible for inclusion if they were aged 70 years or above, presented at the ED with a suspected hip fracture after falling from a standing position, received imaging which did not show a fracture and were subsequently diagnosed with a hip contusion. Patients with previous hip surgery were excluded due to prior positive or negative experiences with the rehabilitation process. Patients were divided in three cohorts based on frailty criteria and the presence of a pre-existing diagnosis of dementia. Patients were considered frail if they met one of the frail hip criteria of Loggers et al.; BMI lower than 18.5, and/or a pre-trauma Functional Ambulation Category (FAC) score of 2 or lower, and/or American Society of Anaesthesiologists (ASA) score of 4 or 5 [6]. Patients in cohort A contained patients who did not meet the frailty criteria (hereafter referred to as ‘non-frail’), cohort B contained patients who did meet the frailty criteria (hereafter referred to as ‘frail’) and cohort C contained patients with a pre-existing diagnosis of dementia. Due to the presence of a pre-existing diagnosis of dementia in patients in cohort C, the questionnaire was conducted in proxies. Convenience sampling was used, where only patients who were known in one hospital were contacted. All participants gave informed consent before conducting the structured interview.

### Outcome parameters

The primary outcome was the ranking of the most important GOC in all cohorts. In the structured interview, participants were asked to indicate the relative importance of GOC on a 100-point scoring scale, from 0 (totally unimportant) to 100 (utmost importance). GOC were ranked using medians, with the highest median classified as the most important GOC. The medians were deemed most important for the ranking of GOC. However, in the boxplots, we displayed equal GOC with a higher third quartile visually higher in the ranking when medians were similar. A GOC was considered most important when scored with a median of 90 or higher. A secondary outcome was the extent to which our participants had engaged in ACP. This required asking, by means of affirmative questions, the level of ACP in patients.

### Data collection

TN and DL conducted the interviews in October 2022. Both authors (TN and DL) had prior experience in conducting interviews and qualitative research. The answers of the participants were coded and uploaded to a secure server with a key accessible to TN and DL. Patient characteristics were collected from the electronic medical records: including age (in years), sex, presence of a pre-existing diagnosis of dementia diagnosed by a physician, living situation (independent at home, home with activities of daily living care, institutional care facility), Charlson Comorbidity Index (CCI), body mass index (BMI), Functional Ambulation Category (FAC) and American Society of Anaesthesiologists Classification (ASA). From the proxies, data were collected during the interview including age (in years), sex and relationship with the patient (spouse, partner, offspring, and caregiver).

### Data analysis

Continuous data were reported as median and interquartile ranges (IQR) due to non-normal distribution. In absence of relative weights attached to the possible outcomes after a hip fracture, a formal power analysis was considered inappropriate, therefore, no statistical comparison was made between GOCs. A pragmatic sample size of 20 participants in each cohort was deemed sufficient to achieve data saturation. For each participant, the range between the highest and lowest score was calculated, to assess dispersion in the valuation by individual participants.

## Results

**Fig. 1:**
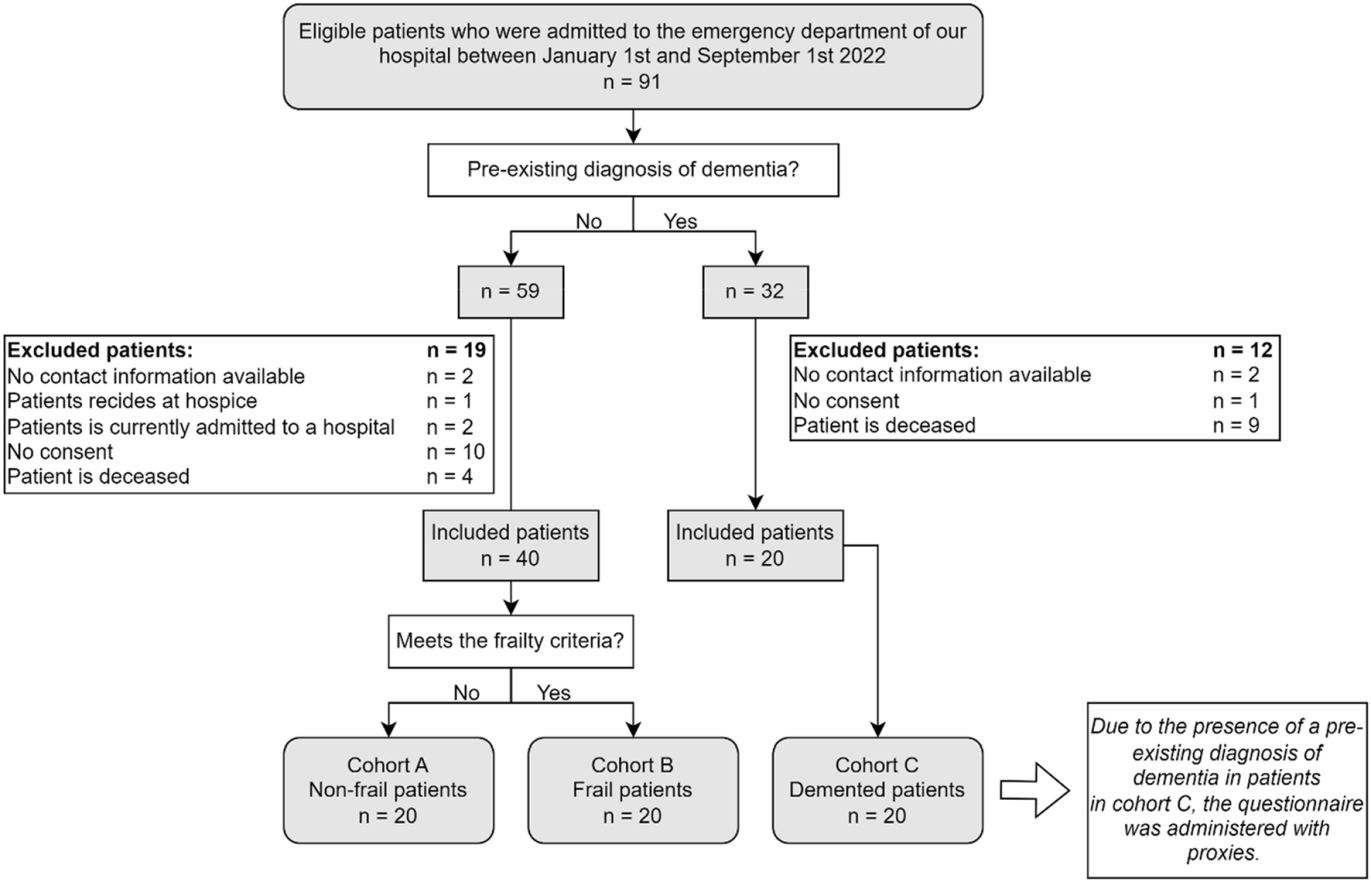
Flowchart of selection process of study population. Frailty criteria = BMI lower than 18.5, and/or a Functional Ambulation Category (FAC) score of 2 or lower pre-trauma and/or American Society of Anaesthesiologists (ASA) score > 3 [6]. Cohort A = non-frail patients with no pre-existing diagnosis of dementia. Cohort B = frail patients who meet the frailty criteria of Loggers et al. with no pre-existing diagnosis of dementia. Cohort C = patients with a pre-existing diagnosis of dementia, where the interview was conducted by proxies

Of the 91 eligible patients, 60 patients were included, 20 in each cohort (Fig. 1). All interviews were conducted within 18 months after presentation at the ED with a hip contusion. The included patients had a median age of 83 (IQR 78–88) years, 42 patients (70%) were female with a median CCI of 5 (IQR 4–7) and 22 patients (37%) lived in an institutional care facility (Table 1). In cohort B and C, respectively, 20 and 19 patients met the frailty criteria. In total 39 (65%) eligible patients met the frailty criteria, 38 based on their functional ambulation category and one patient with a BMI lower than 18.5. Overall, 50 (83%) patients had discussed with their relatives what they would want in the event of an acute illness, and 25 (42%) also discussed treatment preferences when sustaining a hip fracture (Table 2).

**Table 1:** Baseline characteristics of included patients and proxies

Patient characteristics				
	Total (*n* = 60)	Cohort A Non-frail patients (*n* = 20)	Cohort B Frail patients (*n* = 20)	Cohort C Demented patients (*n* = 20)
Age (Y), median (IQR)	83 (78–88)	78 (75–83)	86 (78–89)	85 (83–93)
Female sex, *n* (%)	42 (70)	9 (45)	14 (70)	19 (95)
Pre-existing dementia, *n* (%)	20 (33)	0 (0)	0 (0)	20 (100)
Living situation, *n* (%)				
Home, independent	19 (32)	16 (80)	3 (15)	0 (0)
Home, with ADL care	19 (32)	4 (20)	13 (65)	2 (10)
Institutional care facility	22 (37)	0 (0)	4 (20)	18 (90)
CCI, median (IQR)	5 (4–7)	4 (3–7)	4 (4–6)	6 (5–7)
BMI, median (IQR)	25.6 (21.7–29.6)	25.8 (22.2–29.1)	27.4 (24.1–34.7)	24.4 (20.4–27.7)
FAC, *n* (%)				
FAC 0	4 (7)	0 (0)	0 (0)	4 (20)
FAC 1	12 (20)	0 (0)	3 (15)	9 (45)
FAC 2	23 (38)	0 (0)	17 (85)	6 (30)
FAC 3	3 (5)	2 (10)	0 (0)	1 (5)
FAC 4	8 (13)	8 (40)	0 (0)	0 (0)
FAC 5	10 (17)	10 (50)	0 (0)	0 (0)
ASA, *n* (%)				
ASA 1	2 (3)	1 (5)	1 (5)	0 (0)
ASA 2	14 (23)	6 (30)	4 (20)	4 (20)
ASA 3	44 (73)	13 (65)	15 (75)	16 (80)
Meets the frailty criteria^a^, *n* (%)	39 (65)	0 (0)	20 (100)	19 (95)
BMI lower than 18.5	1 (2)	0 (0)	0 (0)	1 (5)
FAC 2 or lower	38 (63)	0 (0)	20 (100)	18 (90)
ASA 4 or higher	0 (0)	0 (0)	0 (0)	0 (0)

Proxy characteristics				
Age (Y), median (IQR)	–	–	–	59 (54–62)
Female sex, *n* (%)	–	–	–	14 (70)
Relationship with patient, *n* (%)				
Offspring	–	–	–	20 (100)

All variables are in total amount (percentage) or median (interquartile range, IQR)

Frailty criteria^a^ = BMI lower than 18.5, and/or a Functional Ambulation Category (FAC) score of 2 or lower pre-trauma and/or American Society of Anaesthesiologists (ASA) score > 3[6]

*Y* years, *ADL* activities of daily living, *BMI* body mass index, *FAC* functional ambulation classification, *ASA* American Society of Anesthesiologists Classification, *CCI* Charlson Comorbidity Index [36]

**Table 2:** The extent of advance care planning in geriatric patients

	Total (*n* = 60)	Cohort A Non-frail patients (*n* = 20)	Cohort B Frail patients (*n* = 20)	Cohort C Demented patients (*n* = 20)
ACP, *n* (%)				
“I have thought about treatment options for when I become very ill”	50 (83)	15 (75.0)	17 (85.0)	18 (90.0)
“I shared my thoughts with my surroundings”	43 (72)	10 (50.0)	15 (75.0)	18 (90.0)
“I have thought about treatment options for when I sustain a hip fracture”	25 (42)	6 (30.0)	9 (45.0)	10 (50.0)

All variables are in total amount (percentage)

*ACP* advance care planning

### Validity and completeness

Overall, the validity statement was scored 50 points lower on average than the QoL statement with a median score of 90 (IQR 80–100) on the QoL and the validity statement with an overall median score of 40 (IQR 20–60). All answers on the validity statement were deemed sufficient to include the interviews in the analysis. After completing the interview, all participants indicated no additional or missing goals of care in the questionnaire.

### Non-frail patients (cohort A)

**Fig. 2:**
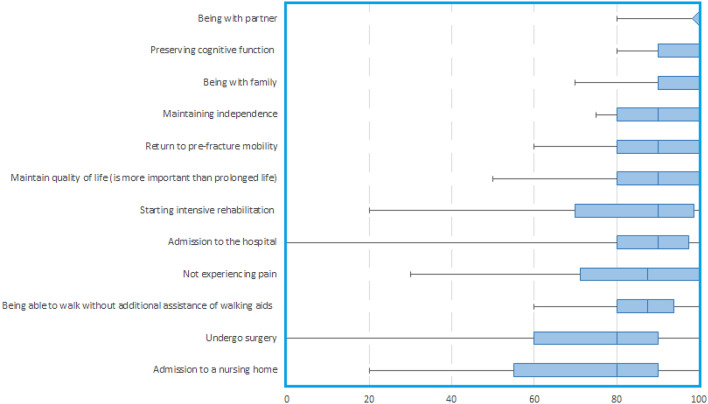
Boxplot of Goals of Care of non-frail, patients (cohort A). 0 = totally unimportant. 100 = utmost importance

Non-frail patients in cohort A had a median age of 78 (IQR 75–83) years, 9 patients (45%) were female and 18 patients (80%) lived independently at home (Table 1). Median scores for the GOC are presented in Table 3. For non-frail geriatric patients, eight GOC were ranked as most important (with a median score of 90 or higher): being with partner 100 (IQR 100–100), preserving cognitive function 100 (IQR 90–100), being with family 100 (IQR 90–100), maintaining independence 90 (IQR 80–100), return to pre-fracture mobility 90 (IQR 80–100), maintain QoL 90 (IQR 80–100), starting intensive rehabilitation 90 (IQR 70–99) and admission to the hospital 90 (IQR 80–98) (Fig. 2).

**Table 3:** Ranking of goals of care for all cohorts

	Cohort A Non-frail patients (*n* = 20)		Cohort B Frail patients (*n* = 20)		Cohort C Proxy-reported (*n* = 20)	
	Median	Rank	Median	Rank	Median	Rank
Treatment of hip fracture						
Not experiencing pain	88 (71–100)	9	83 (63–98)	9	100 (96–100)	1
Admission to the hospital	90 (80–98)	8	83 (71–98)	8	55 (13–80)	10
Undergo surgery	80 (60–90)	11	70 (50–88)	12	28 (10–50)	12
Return to pre-fracture mobility	90 (80–100)	4^a^	90 (73–100)	5	70 (43–90)	8
Rehabilitate						
Maintaining independence	90 (80–100)	4^a^	90 (80–100)	4	75 (50–88)	7
Being able to walk without additional assistance of walking aids	88 (80–94)	10	83 (63–100)	7	80 (53–90)	6
Starting intensive rehabilitation	90 (70–99)	7	78 (61–90)	10	50 (19–75)	11
Admission to a nursing home	80 (55–90)	12	75 (53–90)	11	55 (21–80)	9
Quality of life						
Maintain quality of life (is more important than prolonged life)	90 (80–100)	4^a^	83 (80–100)	6	100 (75–100)	2
Preserving cognitive function	100 (90–100)	2^a^	100 (90–100)	1	93 (76–100)	5
Being with family	100 (90–100)	2^a^	98 (85–100)	3	98 (83–100)	4
Being with partner	100 (100–100)	1	100 (84–100)	2	100 (60–100)	3
Validation question						
Length of life is more important than quality of life	50 (33–68)		50 (33–60)		30 (16–40)	

Outcomes are in median (interquartile range, IQR)

Frailty criteria = BMI lower than 18.5, and/or a Functional Ambulation Category (FAC) score of 2 or lower pre-trauma and/or American Society of Anaesthesiologists (ASA) score > 3[6]

^a^Ex aequo

### Frail patients (cohort B)

**Fig. 3:**
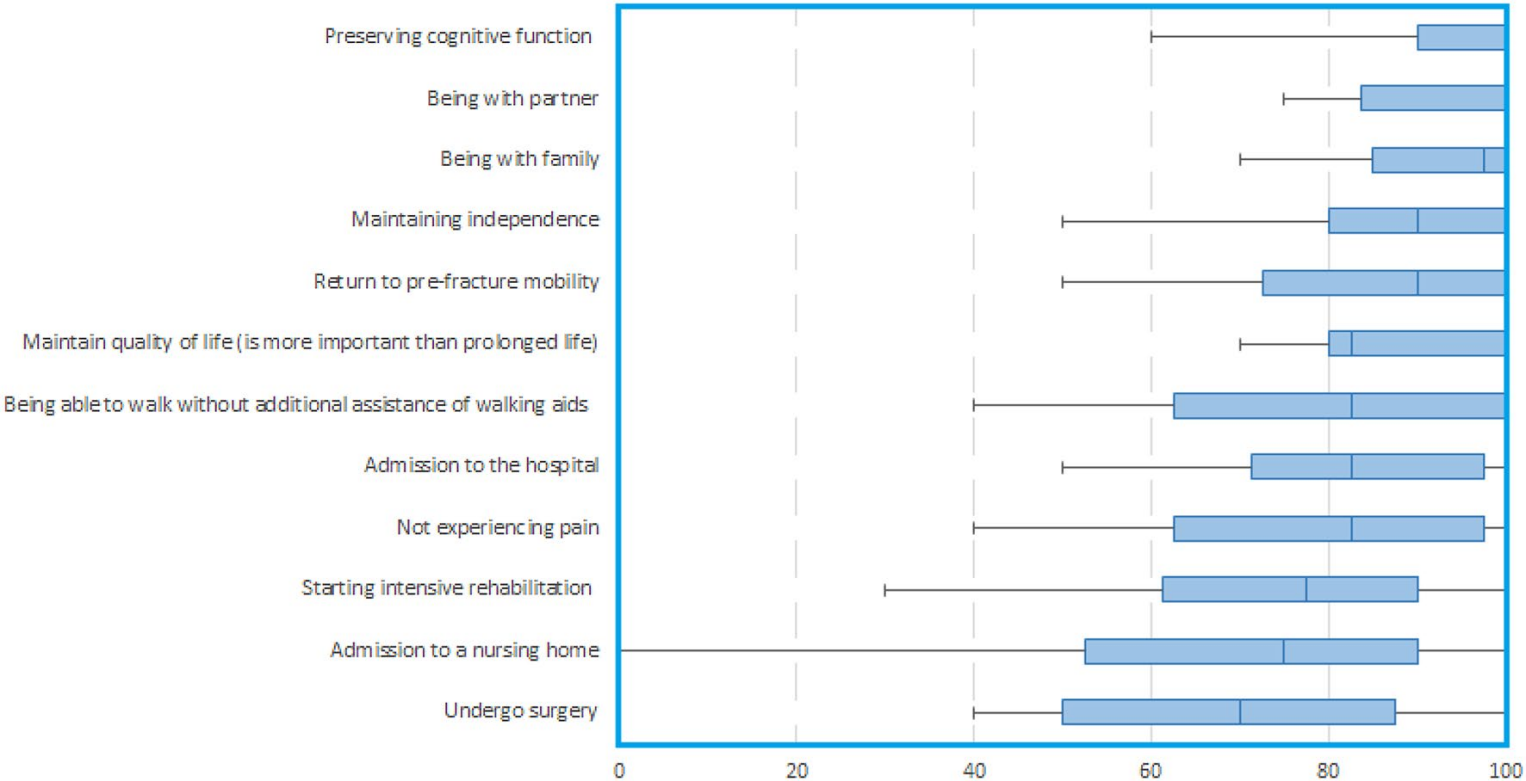
Boxplot of goals of care of frail, patients (cohort B). 0 = totally unimportant. 100 = utmost importance

Frail patients in cohort B had a median age of 86 (IQR 78–89) years, 14 patients (70%) were female and 16 patients (80%) lived at home including 13 (65%) patients of whom received additional ADL care (Table 1). Median scores for the GOC are presented in Table 3. For frail geriatric patients, five GOC were ranked as most important (with a median score of 90 or higher): preserving cognitive function 100 (IQR 90–100), being with partner 100 (IQR 84–100), being with family 98 (IQR 85–100), maintaining independence 90 (IQR 80–100), and being able to walk again 90 (IQR 73–100) (Fig. 3).

### Proxies of patients with a pre-existing diagnosis of dementia (cohort C)

**Fig. 4:**
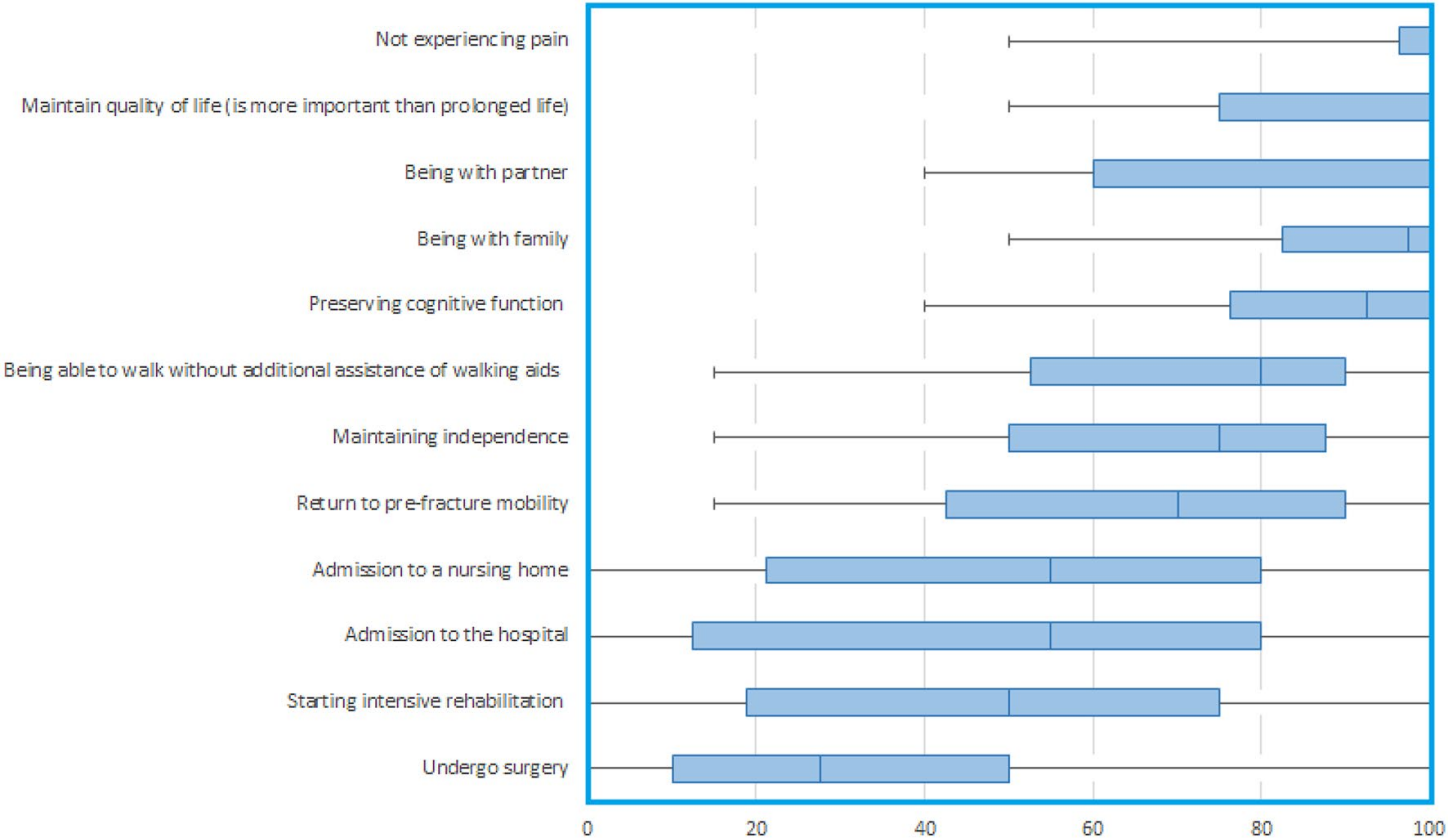
Boxplot of proxy-reported goals of care of patients with a pre-existing diagnosis of dementia (cohort C). 0 = totally unimportant. 100 = utmost importance

Patients with a pre-existing diagnosis of dementia in cohort C had a median age of 85 (IQR 83–93) years, 19 patients (95%) were female and 18 (90%) of patients lived in an institutional care facility. Proxies interviewed were all offspring from the included patients. Proxies had a median age of 59 (IQR 54–62) years and 14 proxies (70%) were female (Table 1). Median scores for the GOC are presented in Table 3. For proxies of patients with a pre-existing diagnosis of dementia, five GOC were ranked as most important (with a median score of 90 or higher): not experiencing pain 100 (IQR 96–100), maintain QoL 100 (IQR 75–100), being with partner 100 (IQR 60–100), being with family 98 (IQR 83–100), and preserving cognitive function 93 (IQR 76–100) (Fig. 4).

## Discussion

A cross-sectional survey study was conducted to obtain the most important GOC for geriatric patients in hip fracture setting. Non-frail geriatric patients, frail geriatric patients and proxies of geriatric patients with a pre-existing diagnosis of dementia all scored preserving cognitive function, being with family and being with partner among the most important GOC. Both non-frail and frail geriatric patients scored return to pre-fracture mobility and maintaining independence among the most important GOC, where proxies of patients with a pre-existing diagnosis of dementia scored not experiencing pain as the most important GOC. These most important GOC may guide a physician at the ED, ultimately allowing SDM to be more efficient for these complicated patient populations.

Preserving cognitive function scored high across all cohorts, with non-frail and frail patients scoring it with a median of 100 and proxies scoring it with a median of 93. This reflects the results of Steinhauser et al., who described being mentally aware as highly important for patients [24]. Since patients value preserving cognitive function so highly, it is import to inform patients during SDM that there is a risk of cognitive dysfunction after surgery [25–27]. ‘Undergo surgery’ is scored relatively low compared to the other GOC, with frail, geriatric patients considering hip fracture surgery as least important. This corresponds with recent studies describing that GOC of the frailest patients focus more on QoL and comfort rather than physical performance [5, 28]. Similarly, proxies of patients with dementia scored hip fracture surgery the least important. Also, a recent study investigating the public’s opinion on life-sustaining treatment supported this finding, in which the majority (68.9%) of the participants wanted no life-sustaining treatment for their partners in the case of dementia [28]. Relatives described adverse affection regarding suffering, decay, or pain for their demented loved ones which probably explains why life prolonging surgery is considered least important by these proxies [29, 30]. With regards to pain, proxies scored not experiencing pain the most important GOC, with a median of 100. A recently published qualitative study into the proxy-reported experiences of palliative, non-operative management, supports proxies valuing being pain free most important for the patients comfort [8]. Remarkably, not experiencing pain is only ranked 9th by non-frail and frail geriatric patients, with a median score of, respectively, 88 and 83, while other studies have emphasized the importance of being pain free to maintain QoL [24, 29]. Patients clearly underlined in the interviews that pain in acute setting was expected in case of trauma or fracture and therefore not deemed most important. However, for proxies seeing their loved ones in pain could be unbearable, resulting in higher scores. In this study, 12 GOC were identified for geriatric hip fracture patients. This discriminates us from the Outcome Prioritization Tool, which was developed for geriatric patients in general. In the Outcome Prioritization Tool, patients rank four health outcomes: extending life, maintaining independence, reducing pain and reducing other symptoms [14]. Recent studies showed that the majority of patients ranked maintaining independence as most important [30–32]. In this study, maintaining independence was the fourth most important GOC. However, the Outcome Prioritization Tool did not include the top 3 GOC of this study (‘being with partner’, ‘maintaining cognition’ and ‘being with family’). Geriatric patients ranked admission to a nursing home as least or second-least important GOC. Several studies have shown health expectancies of geriatric patients can be strongly influenced by several factors, for example health status of peers suffering from worse health [33–35]. Therefore, it is tempting to speculate geriatric patients do not want to be admitted to a nursing home because they consider themselves vital and independent enough returning home instead of receiving nursing care.

One of the strengths of this study was that the composition of the GOC was developed by a multidisciplinary team consisting of four physicians with different geriatric focus areas and all directly involved in the management of geriatric hip fracture patients. In addition, the GOC were patient-centered and pretesting ensured that the interview was complete and comprehensible. A hypothetical situation regarding a hip fracture was presented without the need of recalling information from the past to avoid any forms of recall bias. The validity statement showed patients understood the questionnaire. Patients reported no missing GOC at the end of the interview, indicating a good content validity of the questionnaire. The inclusion of hip contusion instead of hip fracture patients could be a limitation for assessing GOC. Interviewing hip fracture patients in the short interval between arrival at the emergency department and the moment when shared decision-making takes place was deemed undesirable for conducting an interview. Patients with a hip contusion were deemed as the most appropriate study population because this population is similar in characteristics to hip fracture population and cannot be influenced by experiences with OM [21]. Convenience sampling could introduce bias, where only patients who were known in one hospital were included. Since the hospital had a trauma geriatric unit, potential participants and date were relatively easily accessible with little missing data. To minimize the possibility of bias from our sampling strategy, all patient were deemed eligible for inclusion within a time span of 18 months. The last possible limitation is that geriatric patients without a diagnosis of dementia could have signs of mild cognitive impairment, this study did not include cognitive assessment during the interviews and only a validity statement was included to assess comprehension of the interview.

The results of this study are directly applicable in clinical practice. Since the decision for operative management is usually made within a short period of time, implementation of these GOC during the pre-operative SDM process will allow further validation of their individual importance for each patient category. The post-operative period following hip fracture surgery may be complicated by cognitive dysfunction, therefore the possibility of cognitive decline should always be discussed with patients undergoing surgery. Since there is a discrepancy between the ranking of not experiencing pain between geriatric patients and proxies, proxies could be better informed about the expected hip fracture related pain of their relatives.

## Conclusion

This study explored the most important GOC for geriatric patients in hip fracture setting. Non-frail patients, frail patients and proxies of patients with dementia all scored preserving cognitive function, being with family and being with partner among the most important GOC. The most important GOC should at least be discussed when a patient is presented at the ED with a hip fracture. In addition, since patients preferences vary, a patient-centered assessment of the GOC remains essential during SDM. Future research should perform statistical intercohort differences in GOC for hip fracture patients which could result in even more targeted tailor-made SDM discussions.

## Data Availability

Data are available on reasonable request.

## Appendix 1: Structured interview

Abbreviations

Q = Question

O = Outcome

S = Statement

GOC = Goal of Care

“First, A few general questions follow. You may answer with yes or no.

### Questions regarding advanced care planning

Q1: Did you think about what you would want to happen tomorrow you suddenly become very sick?

Q2: Did you share the above with your family, or with your family doctor?

Q3: Did you think about what you would want to happen in the case you sustain a hip fracture

“Now try to imagine coming back to the emergency room after a fall, and now you do have a broken hip. I will list 13 possible outcomes after a broken hip. Please rate on a scale of 0-100 how important you think these outcomes are. A 0 can be given if the outcome is not important to you at all, and 100 should be given to the most important outcome according to you.”

### Questions regarding possible outcomes after surgery

GOC1: Not experiencing pain

S1: Experiencing no pain

GOC2: Admission to the hospital

S2: Become admitted to a hospital

GOC3: Undergo surgery

S3: Undergo hip fracture surgery, with the risk of complications

GOC4: Return to pre-fracture mobility

S4: Return to pre-fracture mobility, even if I need surgery to do so

### Questions regarding rehabilitation

GOC5: Maintaining independence

S5: Maintain independence

GOC6: Being able to walk without additional assistance of walking aids

S6: To be able to walk as long as possible without additional aids

GOC7: Starting intensive rehabilitation

S7: Start intensive rehabilitation to return to pre-fracture situation.

GOC8: Admission to a nursing home

S8: Rehabilitation, even if it means admission to a nursing home temporarily

### Questions regarding quality of life

GOC9: Maintain quality of life (is more important than prolonged life)

S9: Maintain quality of life is more important than the length of my life.

GOC10: Preserving cognitive function

S10: Maintain cognition

GOC11: Length of life is more important than quality of life

S11*: Prolonged life is more important than quality of life

* = validation question, the opposite of statement 9

GOC12: Being with family

S12: To have final moments with family regardless of the length of my life.

GOC13: Being with partner

S13: I would like to stay together with my partner

### Final question:

After rating all these goals of care, did you miss any important goals of care in this interview?

If so, what goal of care did you miss, and how important is it to you?
